# Supplementary material for: Use of a cooling pack to reduce subcutaneous vaccine injection pain in children aged 3–6 years: A single-blind, randomized, parallel-group multicenter study
Source: PLoS One. 2025 Mar 26;20(3):e0318322. doi: 10.1371/journal.pone.0318322 (PMC11940606; doi:10.1371/journal.pone.0318322)
Supplement: S2 File — (DOCX) [file pone.0318322.s002.docx]

Trial protocol

Effectiveness and safety of cooling pack TAK-01

for relieving pain at subcutaneous injection site in young children

Principal Investigator

Ikuo Okafuji

Department of Pediatrics, Kobe City Medical Center General Hospital

| The information in this document is provided only to those directly involved in this study (e.g., administrators of the implementing medical institution, research administration, principal investigators, principal investigators or subinvestigators, research subjects, study device administrators, and authorized clinical research review committees). Therefore, information may not be disclosed to third parties unrelated to this study without the prior consent of the principal investigator, except when consent is obtained from the subjects participating in the study. |
| --- |

Research Protocol No. TAK-01

Edition 1.1

Creation Date February 1, 2021

TABLE OF CONTENTS

1. Outline of Clinical Research .............................................................................................................. 4
2. Background......................................................................................................................................... 5
3. Objective.............................................................................................................................................. 7
4. Outline of TAK-01................................................................................................................................ 7
5. Research Subjects................................................................................................................................ 8
6. Clinical Research Methods ................................................................................................................. 8
7. Assessment Item................................................................................................................................ 11
8. Observation and examination items................................................................................................ 11
9. Criteria for discontinuance............................................................................................................... 13
10. Handling of Adverse Events and Diseases, etc. .............................................................................. 14
11. Handling of defects............................................................................................................................ 15
12. Initiation, Suspension, Discontinuation and Termination of Clinical Research ……………...... 16
13. Clinical Research Period.................................................................................................................... 17
14. Analysis targets and statistical analysis methods........................................................................... 17
15. Changes in research protocols, procedures, or statistical analysis plans, etc………………………… 19
16. Data Management.............................................................................................................................. 19
17. Quality Control and Quality Assurance............................................................................................ 20
18. Compliance with the Clinical Research Act and the Declaration of Helsinki, etc. …………………… 21
19. Consideration for human rights and safety and disadvantage of research subjects …………….… 21
20. How to explain to research subjects and obtain their consent ..................................................... 22
21. Compensation for health damage..................................................................................................... 23
22. Cost-sharing of research subjects..................................................................................................... 23
23. Handling and Preservation of Records, etc...................................................................................... 24
24. Registration of research plan............................................................................................................ 24
25. Sources of Research Funding and Conflict of Interest Status......................................................... 25
26. Attribution of Research Results and Publication of Research Results........................................... 25
27. Report to CRB, etc. ....................................................................................................../...................... 26
28. Research Organization...................................................................................................................... 26
29. References ......................................................................................................................................... 26
30. Revision History................................................................................................................................ 27

List of abbreviations

| abbreviation | Unabbreviated Expression (English) | Unabbreviated expressions (Japanese) |
| --- | --- | --- |
| FLACC | Face, Legs, Activity, Cry, Consolability Scale | no translation into Japanese |

1. Outline of Clinical Research

| Objective. | Exploring the pain-relieving effects and safety of TAK-01 when injected subcutaneously in young children |
| --- | --- |
| Test Equipment | TAK-01 (cooling pack) |
| Research Design | Multicenter, two-arm, single-blind, randomized, parallel-group study |
| person who is being studied | Preschool children aged 3 to 6 at the time of obtaining consent to receive immunization by subcutaneous injection |
| Primary Endpoint | FLACC scale evaluation by FLACC scale evaluation committee members after immunization implementation |
| secondary endpoint | FLACC scale evaluation after immunization by attendant  Incidence of Adverse Events |
| Eligibility Criteria | Selection Criteria] The following criteria are all met at the time of case enrollment.   1. Preschool children aged 3 to 6 years at the time of case enrollment 2. Those scheduled to receive subcutaneous Japanese encephalitis vaccine or influenza vaccine at the time of case enrollment. 3. A person who has obtained the free and voluntary written consent of a surrogate to participate in this study.   Exclusion Criteria] Patients who meet at least one of the following criteria at the time of case registration are eligible  Exclude as   1. Those who are chirping more intensely than before consent was obtained. 2. Persons with sensory insensitivity or intellectual disability at the time of obtaining consent 3. Persons scheduled to receive two or more vaccinations on the date consent is obtained 4. Who used analgesics within 6 hours prior to case enrollment 5. Persons diagnosed with or suspected of having a cold-stimulus-related illness such as Raynaud's syndrome or sickle cell disease at the time consent is obtained   (6) Persons who are judged by the principal investigator (subinvestigator) to be inappropriate to participate in this study |
| Observation Schedule | Obtain consent, assign, and use the test device (hereafter referred to as TAK-01) at the time of vaccination outpatient visit, and observe until 10 minutes after the vaccination is administered. |
| Target number of cases | 60 cases (30 in cooling group, 30 in non-cooling group) |
| Scheduled implementation period | April 2021 - September 2022  (Patient enrollment period: jRCT release date to March 2022) |

#

2. Background

Medical procedures on children, including injections, typically cause pain and distress. Relieving pain during medical procedures promotes a positive attitude toward later medical care(1) . According to evidence-based clinical practice guidelines, a combination of pharmacologic, physical, and psychological approaches is effective in reducing pain in children from immunizations(2) (3) (4) (4) (5) (6) (7) (8) (9)

A network meta-analysis of randomized controlled trials examining the effect of pharmacological interventions on vaccine pain relief in children concluded that topical application of a lidocaine and procaine eutectic mixture cream formulation (Emla Cream®) significantly relieved pain, although the level of evidence was insufficient(4) . In one of the studies examined in this analysis, a double-blind, randomized, controlled trial of the pain-relieving effect of Emla Cream® at the time of vaccination in young children (4-6 years), using the affected children's own face pain scale (0 = no pain, 6 = maximum pain on a 7-point scale), the intervention group (n = 83, median 1.3 , SD 1.87) showed significantly greater pain relief than the placebo group (n=76, median 2.3, SD 2.31) (p=0.02)(5). In Japan, Hanaoka et al. studied the local anesthetic effect of Emla cream at the time of venipuncture in adults, but not in adults between 20 and 65 years of age, in a multicenter, placebo-controlled, randomized, double-blind, parallel-group study with the Visual Analogue Scale (VAS) of pain as the primary endpoint showed a significant local anesthetic effect in the intervention group (n=42, median 18.88 mm, SD 17.36) compared to the placebo group (n=44, median 39.6 mm, SD 22.94) (p < 0.001).(6) .

The World Health Organization's position paper on pain relief at the time of vaccination considers interests, values and preferences of the vaccinated, resource utilization, intervention costs, equity implications, acceptability, and feasibility from a global perspective, and outlines the following four policies(7). The following four policies have been identified in consideration of the value of the individual being vaccinated: (i)  Health-care personnel carrying out vaccination should be calm, collaborative and well informed; they should use neutral words (e.g. “here I go” rather than “here comes the sting”) and avoid language that increases anxiety, promotes distrust and/or is falsely reassuring or dishonest (e.g. phrases such as “it will only hurt for a second”). (ii)  Proper positioning of the vaccine recipient should be ensured, according to age.8 Holding by the care- giver is recommended for infants and young children, and sitting upright for older populations. Lying down may be preferred for those with a history of fainting. (iii)  No aspiration should be done during intramuscular injections, as this may increase pain due to longer contact time and lateral movement of the needle. (iv) When multiple vaccines are injected sequentially in the same session they should be administered in order of increasing painfulness. The following four special considerations should be made for children:(i) The caregiver should be present throughout and after the vaccination procedure. (ii)  Infants and children aged <3 years should be held by caregivers throughout the procedure, and those aged ≥3 years should be seated to alleviate fear and distress, preferably on the caregiver’s lap. (iii)  If culturally acceptable, breastfeeding of infants should be done during or shortly before the vaccination session.11 Where oral vaccines are being co-administered with injectable vaccines, it would be best to proceed with the administration of oral rotavirus vaccine, then oral polio vaccine (if OPV is used), then breastfeeding with simultaneous administration of the injectable vaccines. (iv)  For children <6 years of age, distractions to divert attention away from pain to something more pleas- ant (e.g. with toys, video, music, or conversation with an adult) are recommended. In our country, pharmacological interventions are rarely used at the time of vaccination, although there are opportunities to use them for planned procedures for chronically ill pediatric patients. In fact, the accompanying dosage and administration of Emla Cream® states that the cream should be applied 60 minutes before the procedure is performed, which takes time for the effect to develop.

On the other hand, skin cooling has been used for centuries to relieve pain. Skin cooling is characterized by its rapid onset of pain relief, simplicity, cost-effectiveness, and virtually no side effects. The pain-relieving effect of skin cooling begins to appear at a skin temperature of 10°C and increases as the temperature approaches 0°C. The analgesic effect is also enhanced by the suppression of autonomic nervous system responses due to the decrease in skin conductivity and skin blood flow caused by cooling. In actual clinical practice, we have been cooling the inoculation site before inoculation for infants who refuse subcutaneous injections because they complain of pain from subcutaneous injections. We have had many cases in which infants who refused subcutaneous injections were well accepted, and we were able to elicit a positive attitude toward subcutaneous injections. Initially, ice packs were made by filling medical gloves with ice water, but since several years ago, we have been using "PuruCURE Ice Pack®," a cooling pack manufactured and marketed by Mie Chemical Industry Co. However, a systematic review of the pain-relieving effects of vaccination site skin cooling has shown that it is effective in adults, but no conclusion can be drawn at this time for children(8) . The reasons for this may include the fact that children are less expressive of their emotions than adults, and that in many papers, the pain assessors are not the patients themselves but their parents or guardians. Therefore, we decided to investigate the pain-relieving effects and safety of TAK-01 during subcutaneous injection for infants by having the currently marketed "PuruCURE Icepack®" manufactured in a smaller size that is easier to apply to infants' upper arms to improve convenience.

3. Objective

Exploring the pain-relieving effects and safety of TAK-01 when injected subcutaneously in young children

4. Outline of TAK-01

4.1 TAK-01 shape, structure and principle

　 Structure


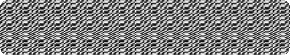

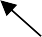


　　Coolant contents (gel) Crystal Gel®* (gel)

*Crystal Gel® will be used by Tanac Corporation, and Mie Chemical Co., Ltd. will handle the rest of the sales, including filling of coolant contents.


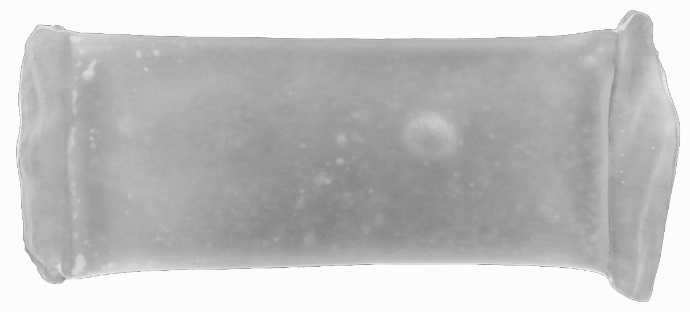
Shape

**95 mm**

**135 mm**

The size of about 95 mm x 270 mm is called "Puru CURE Icepack®" and is sold under the trade name "Puru CURE Icepack®.

Notified as a general medical device by Mie Chemical Industry Co.

Classification: Instrument 12 Physiotherapy equipment General name: Cooling pack (Code: 37240020)

　　　Principle

　　　　　This product containing a cooling medium is cooled and stored in a cooling device, and the cooling effect is used to cool the affected area.

Cooling

4.1.2 TAK-01 providers

Mie Chemical Industry Co.

4.2 Anticipated diseases, etc.

Frostbite, etc. when used directly against the skin.

4.3 Anticipated Defects

Damage due to excessive load

5. Research Subjects

5.1 Selection Criteria

The study will include those who meet all of the following criteria at the time of case enrollment

1. Preschool children aged 3 to 6 years at the time of case enrollment
2. Those scheduled to receive subcutaneous Japanese encephalitis or influenza vaccination at the time of case enrollment.
3. A person who has obtained the free and voluntary written consent of a surrogate to participate in this study.

Basis for setting

　(1) Provisions pertaining to the subject of this study, in order to reduce the risk of a blind split.

　(2) For regulations pertaining to the subject of this research

(3) To protect research subjects

5.2 Exclusion Criteria

At the time of case enrollment, patients with any one of the following conditions will be excluded from the study

1. Those who have been chirping violently since before consent was obtained
2. Persons with sensory insensitivity or intellectual disability at the time of obtaining consent
3. Persons who are scheduled to receive two or more vaccinations on the date consent is obtained
4. Who used analgesics within 6 hours prior to case registration
5. Persons who have been diagnosed or are suspected of having been diagnosed Raynaud's syndrome, sickle cell disease or cold stimulus-related disease at the time consent is obtained
6. Persons who are judged by the principal investigator (or subinvestigator) to be inappropriate to participate in this study.

Basis for setting

1. If the subject is crying violently, there is a high possibility that the local cooling with TAK-01 itself will be rejected and the study will not be completed.
2. (3)(5)(6) To influence the endpoints and to ensure the safety of research subjects

(4) To affect the evaluation items

(6) To ensure the safety of research subjects

6. Clinical Research Methods

- 1. Research Design Overview

6.1.1 Types and Methods of Clinical Research

Multicenter, single-blind, blind assessment, randomized (allocation factor: institution), two-parallel group comparison

6.1.2 Explanation of the relevance of the study design

　　The study was multicenter and single-blind because the technique of pressing TAK-01 to the injection site makes it difficult to blind the investigators.

　Although applying a cooling pack to the vaccination site is not a standard treatment, the comparison was made between the cooling group and the non-cooling group, rather than between the TAK-01 use group and the non-use group, in order to blind the attendants and the FLACC scale evaluation committee members who were to conduct the FLACC scale evaluation.

　Although it is possible that the 　study subjects themselves could be blinded, the age range was limited to 3-6 years to reduce the risk of such a split, and a blind assessment method by the FLACC scale assessors was used for the primary assessment.

　Because the immunization procedure is also considered a pain factor, the facility was used as an allocation factor to divide the patients into two groups within the facility: a cooling group and a noncooling group.

6.1.3 Duration of subject's participation in the study and observation period

　10 minutes from obtaining　consent to completion of immunization

<Schema: Figure 1>


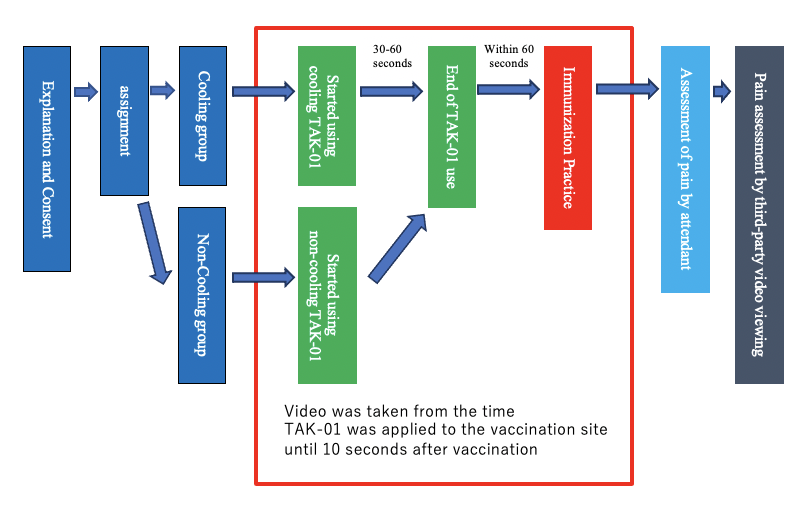


6.2 Registration

6.2.1 Registration Method

　　After explaining this study in writing when the patient comes to the hospital for immunization, the proxy consents to the study.

Registration shall be made upon obtaining and signing the consent form.

6.2.2 Method of Randomization

　After consent is obtained, the investigators at the implementing institution will randomly assign the facility as the allocation factor using the REDCap system.

6.2.3 Blinding procedure

　 The study will be single-blind, with the staff of the implementing medical institution being unblinded and the study subjects and attendants being blinded.

6.3 Research Plan Treatment

　Cooling group: TAK-01 is cooled for at least 3 hours in a freezer (internal temperature: -16 to -20 degrees C), removed from the freezer, and allowed to permeate for 1 to 3 hours in ice water. The TAK-01 is then applied directly to the subject's vaccination site for 30-60 seconds, and inoculation is performed within 1 minute after the TAK-01 is removed.

　Uncooled group: TAK-01 is placed at room temperature (approximately 20 degrees Celsius) for at least 3 hours, and is applied directly to the subject's vaccination site for 30 to 60 seconds.

6.4 Concomitant medications/adjunctive therapy

　Concomitant therapy and concomitant medications

None in particular.

　　 Prohibited concomitant medications and prohibited concomitant therapies

　　　The following concomitant medications/adjunctive therapies are not performed due to their strong impact on the FLACC scale assessment.

　　　　　Prohibited concomitant medications: administration of analgesics and sedatives at the time of vaccination and local anesthesia at the vaccination site.

Concomitant Prohibited Therapy: showing videos or other actions to distract from injections during immunization

6.5 Criteria for Discontinuation of Research Protocol Treatment

If you are unable to receive the vaccination after obtaining consent.

If the TAK-01 is not available to the research subject, or if the time to apply the TAK-01 is less than 30 seconds.

If the vaccination could not be administered within 1 minute after the TAK-01 was removed.

When it is difficult to observe as prescribed due to adverse events, etc.

When the principal investigator decides to discontinue the study

However, for study subjects using TAK-01, in addition to the 10-minute post-immunization observation, the FLACC scale evaluation will be performed immediately after immunization by an attendant and later by the FLACC scale evaluator after viewing the imaged video.

6.6 TAK-01 Administration and Delivery Procedures

　 TAK-01 should be stored in a secure area and kept separate from other equipment.

Details are set forth in the "Test Equipment Management Procedures".

7. Assessment Items

7.1 Primary Endpoints

FLACC scale evaluation after immunization implementation by the FLACC scale evaluation committee members.

One FLACC scale evaluation committee member will be appointed, and the same committee member will evaluate the FLACC scale throughout the study period.

Do.

<Reason for setting

The FLACC Scale was developed by the University of Michigan in the U.S. (8). With permission from Sandra Merkel, the original author, Yujiro Matsuishi et al. in the Department of Emergency and Intensive Care Medicine, University of Tsukuba, translated the FLACC Scale using the back translation method, and confirmed that the scale has minimal errors between raters (9). Therefore, we decided to use this method in this study as well.

7.2 Secondary endpoints

　FLACC scale evaluation after immunization by attendant

Incidence of Adverse Events

7.3 Judging Criteria

FLACC scale


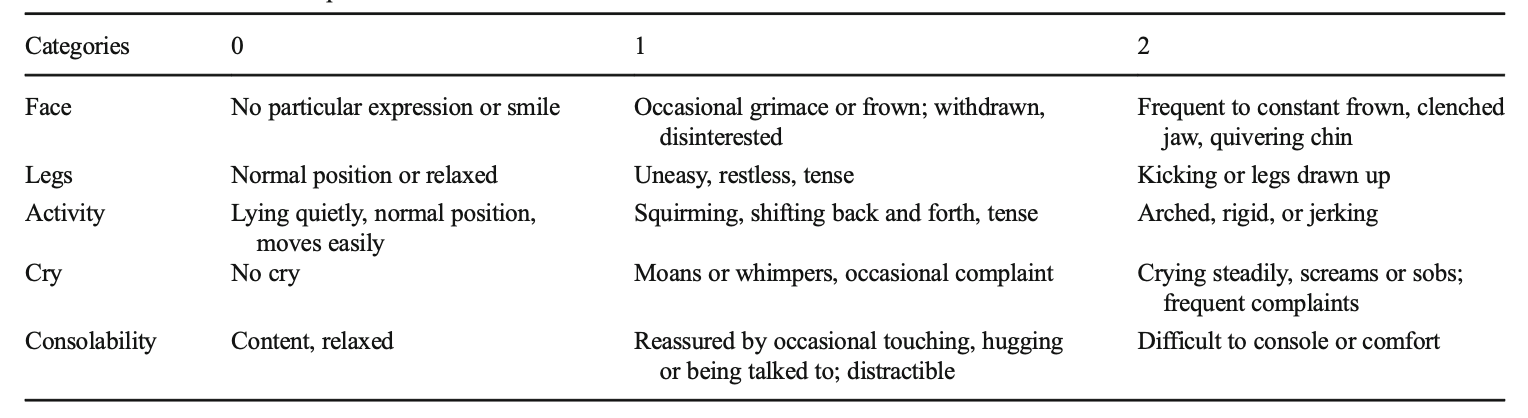
8. Observation and examination items

Schedule Overview

After obtaining written consent from the proxy on the day of vaccination, registration and allocation will be conducted for outpatient immunization recipients of the relevant age group.

Video recording from before TAK-01 use to 10 seconds after inoculation.

The detailed imaging procedures for moving images are set forth in the "Procedures for Imaging".

After vaccination, the FLACC scale was evaluated by an attendant.

The video will be reviewed by the FLACC scale evaluation committee members at a later date and the FLACC scale evaluation will be performed.

The evaluation procedure by the scale evaluation committee members is described in the "Procedures for the Work of the FLACC Scale Evaluation Committee Members".

The following is set forth in Section 2.1.1.

|  | During Outpatient Consultations  Consent/Registration | Before inoculation | vaccination | 10 minutes after inoculation | time of discontinuance |
| --- | --- | --- | --- | --- | --- |
| Basic Information |  |  |  |  |  |
| agreement |  |  |  |  |  |
| Registration and allocation |  |  |  |  |  |
| Video Shooting |  |  |  | ^＊１^ |  |
| Using TAK-01^＊2^ |  |  |  |  |  |
| Immunization Practice^*3^ |  |  |  |  |  |
| FLACC evaluation (by attendant) |  |  |  |  |  |
| adverse event^＊4^ |  |  | | |  |
| Diseases, etc.^＊5^ |  |  |  |  |  |

*1 Movie will be taken 10 seconds before TAK-01 use and after inoculation.

*2 Press the assigned TAK-01 directly on the injection vaccination site for 30-60 seconds

*3 Perform within 1 minute after the end of TAK-01 use.

*4 Adverse events: Collect from the start of application of TAK-01 to 10 minutes after inoculation.

*5 Diseases, etc.: Collect events for which a causal relationship with TAK-01 cannot be ruled out from the start of TAK-01 application to 10 minutes after inoculation.

8.1 During an outpatient visit

8.1.1 Collection of basic information

　　Age, Gender, Types of vaccinations and Blunted sensory perception

8.2 Obtaining Consent and Confirming Eligibility

Obtain written consent from the surrogate and confirm eligibility with the eligibility form.

8.3 allocation

　　Once the eligibility is confirmed on the Eligibility Verification Form, the registration and assignment process is performed in the REDCap system.

8.4 Before inoculation

Started taking video before using TAK-01

Use of TAK-01 in assigned groups

Adverse events, diseases, etc. (collected from the point of use of TAK-01)

8.5 Immunization

　　Collection of adverse events, diseases, etc.

　　　However, adverse events that can be clearly attributed to immunization are not included in the collection.

8.6 After inoculation

　　Video recording ends after 10 seconds of inoculation

　　FLACC scale evaluation by attendant

　　Collect adverse events, diseases, etc. up to 10 minutes after vaccination

8.7 At the time of cancellation

　At the time of discontinuation after use of TAK-01, the following evaluations and data collection will be conducted to the extent possible.

　 　FLACC scale evaluation by attendant

Imaging of immunization status and FLACC scale evaluation by FLACC scale evaluation committee members

Information gathering on adverse events, diseases, etc.

For research subjects not using TAK-01, reasons for discontinuation will be collected to the extent possible.

9. Criteria for discontinuation

If the principal investigator or research associate investigator (hereinafter referred to as "principal investigator, etc.") determines that it is impossible for the research subject to continue participation in the research for any of the following reasons, the research will be discontinued and the timing of the discontinuation or dropout, the reason for the discontinuation or dropout, and the progress, etc. will be noted on the CRF.

9.1 Criteria for Discontinuation of Study Subjects

(1) If the substitute consents to withdraw his/her consent to participate in the clinical research after the commencement of the clinical research

(2) If the research subject is found not to meet the eligibility criteria

(3) If the Principal Investigator, etc. determines that the research subject is unable to comply with the research protocol

(4) Other cases in which the principal investigator determines that the research should be discontinued.

9.2 Post-Cancellation Measures

　At the time of discontinuation, the items listed in 8.7 should be evaluated whenever possible.

10. Handling of Adverse Events, Diseases, etc.

Adverse events and diseases are defined as follows

10.1 Definition of adverse events

All unfavorable events or unintended illnesses or signs of illness (including abnormal laboratory values) that occur in research subjects using TAK-01. This refers to all unfavorable events or unintended illnesses or signs of illness (including abnormalities in clinical laboratory values) that occur in research subjects using TAK-01, regardless of whether they are causally related to this study.

10.2 Serious adverse events

An adverse event is defined as a serious adverse event if any of the following apply

(1) Deadly

(2) May lead to death

(3) Those requiring hospitalization or an extended period of hospitalization for treatment

(4) Obstacles

(5) Threat of failure

(6) Other serious cases according to (1) through (5)

(7) Congenital diseases or anomalies in subsequent generations

10.2.1 Reporting procedures for serious adverse events

(1) If a serious adverse event occurs, the investigator at the site of the study should report the cause and effect of the event to the investigator. Regardless of whether or not there is a relationship, report the occurrence to the principal investigator at the site when you become aware of it.

(2) The principal investigator shall report the occurrence of a serious adverse event to the administrator of the institution using the "Uniform Form 9" and then to the principal investigator as soon as he/she becomes aware of the occurrence of the event.

(3) The Principal Investigator will report the obtained "Uniform Form 9" to the Director of the Principal Investigator's site and then to the Certified Clinical Research Review Board ("CRB"), and provide the information to other principal investigators.

The "Uniform Form 9", which is defined as the reporting form for serious adverse events, shall be used for the reporting of serious adverse events for diseases, etc. as defined in 10.3. This is a reporting form for the occurrence of an event that meets (1) to (7) of 10.2, although it is a reporting form for the occurrence of an event that meets (1) to (7) of 10.2. This is to be used in lieu of the Report Form. The "Uniform Form 9" is used in lieu of the Reporting Form. The report should be supplemented by using the appropriate sections of the standardized form "Form for Detailed Description" for the basis for the determination.

10.3 Definition of Disease, etc.

The period from the use of TAK-01 until the end of observation, including any illness, disability, death, or infection suspected to have resulted from the conduct of this study, as well as any abnormal laboratory values or symptoms. The details of the procedures for reporting the occurrence of illness, etc., as stipulated in the Clinical Research Act, are described in the "Procedures for handling the occurrence of illness, etc.

11. Handling of defects

11.1Definition of defects

This term refers to any problems with the quality, safety, or performance of the TAK-01, such as breakage or malfunction, regardless of whether the problem is caused by the design, delivery, storage, or use of the TAK-01.

If a problem occurs, the research physician must report it to the principal investigator as soon as he or she becomes aware of the problem.

The investigator will report the results to the principal investigator using the "Uniform Form 9. The principal investigator reports to the principal investigator using the "Uniform Form 9.

Failures that should originally be described in the "Uniform Form 9" are only those cases where it is judged that the failure of a medical device used in clinical research may cause disease, etc. However, the "Uniform Form 9" should also be substituted for failures in cases where it is not judged that there is a risk of causing disease, etc.

In addition, any defects that are determined to have the potential to cause health hazards shall be reported in accordance with the "Procedures for handling cases of illness, etc.".

12. initiation, suspension, discontinuation and termination of clinical research

12.1 Initiation of Clinical Research

In conducting this study, the investigator shall prepare an implementation plan (Form 1), attach the necessary documents, and obtain the opinion of the CRB described in said implementation plan. The investigator shall submit said implementation plan to the Minister of Health, Labour and Welfare by registering it in the Japan Registry of Clinical Trials ("jRCT"). The date when the information of the study is registered in jRCT and released to the public shall be the study start date. Consent of research subjects will not be obtained prior to the release of information.

　The principal investigator shall report to the CRB that he/she has submitted said implementation plan to the Minister of Health, Labour and Welfare, which has heard the opinion.

12.2 Criteria for Discontinuation or Suspension of an Entire Clinical Study

　The principal investigator will consider suspending or terminating the entire study if the clinical research meets the following criteria

　(1) In the event of unavoidable ethical or medical circumstances, such as the occurrence of an illness or other event for which a causal relationship to the conduct of this research cannot be denied and for which the risks associated with participation in this research are judged to outweigh the benefits to the research subjects.

　(2) When a serious nonconformity is found in the research protocol, etc.

　(3) If the significance of conducting this research is lost

12.3 Procedures for suspending an entire clinical study

12.3.1 Procedures for Suspension

When suspending the research, the principal investigator shall submit a change in the implementation plan (change in progress) to the CRB and the Minister of Health, Labour and Welfare without delay.

12.3.2 Procedures for reopening

The principal investigator will consider the necessity of revising the research protocol and various procedure manuals before resuming this study after interruption, and will obtain the CRB's opinion. If changes are necessary, they shall be made in accordance with "14. Changes in Research Protocol, Procedures, or Statistical Analysis Protocol, etc.". In addition, the changes in the implementation plan (changes in progress) shall be submitted to the CRB and the Minister of Health, Labour and Welfare without delay.

12.4 Procedures for discontinuation of the entire clinical research

12.4.1 Procedures for Discontinuation

　If the Principal Investigator discontinues this research, he/she shall notify the CRB of the discontinuation (Uniform Form 11) and submit a Notification of Discontinuation of Specified Clinical Research (Ordinance Form 4) to the Minister of Health, Labour and Welfare within 10 days of the date of discontinuation.

The principal investigator shall submit notifications of changes in the implementation plan, disease reports, periodic reports, etc., from the discontinuation of this research until the completion of observation, etc., of all research subjects and the termination of this research.

The Principal Investigator will terminate the study in accordance with "11.5 Termination of Clinical Research" within one year from the later date of either discontinuation of the study or completion of observation of all study subjects.

12.5 Termination of Clinical Research

　The principal investigator will prepare a primary endpoint report within one year after the end of the collection period for the primary endpoints and a summary report and summary report within one year after the end of the collection period for all endpoints.

When the Principal Investigator has prepared a summary of the primary endpoint report or summary report and summary report, he/she shall promptly submit it to the administrator of the implementing medical institution.

13. Clinical Research Period

April 1, 2021 - September 30, 2022

Enrollment period: after publication of jRCT - March 2022

Observation period: from obtaining consent to 10 minutes after vaccination is administered

14. Analysis targets and statistical analysis methods

14.1 Population to be analyzed

14.1.1 largest analysis set (full analysis set: FAS)

The population of study subjects enrolled in the study, for whom subcutaneous injections were administered, and for whom at least one efficacy endpoint was measured, will be the largest population for analysis (FAS).

14.1.2 Subject population conforming to the research protocol (per protocol set: PPS)

The population of patients included in the FAS who are free of material research protocol violations will be defined as the population of subjects (PPS) who conform to the research protocol. Significant study protocol violations will be defined by the principal investigator and the statistical analyst by database fixation after completion of the study.

14.1.3 Population for Safety Analysis

All cases enrolled in the study and treated with TAK-01 will be included in the analysis.

14.2 Target Number of Cases

　Target number of patients: 30 in the cooling group and 30 in the non-cooling group

Rationale: For the primary endpoint of FLACC, a two-sample t-test, assuming an effect size of 0.9, a risk rate of 5% (two-sided) and a power of 90% would require 27 cases in each group. Assuming a dropout rate of about 10% due to inadequate videotaping, etc., 30 cases were needed for each group.

14.3 Data Handling

14.3.1 Data Handling

Abnormal data, including outliers, will be examined before statistical analysis is conducted. In the event that a case deviating from the research protocol is found, the impact of the deviation on the efficacy evaluation of the case will be examined by the principal investigator and the statistical analysis supervisor before the database is fixed after the completion of the study, and the handling of each case will be determined individually.

14.3.2 Handling of Data on Discontinued Research Cases

Data collected up to the time of discontinuation of the study will be used as research data. However, if the consent for the use of the data is withdrawn from the surrogate consentor, the data will not be used.

14.4 Statistical Analysis Items and Analysis Plan

To confirm the effect of pain relief by cooling the subcutaneous injection inoculation site in young children, the FLACC score after subcutaneous injection will be compared with the non-cooling device group as an index. When the null hypothesis (the number of FLACC points at the time of subcutaneous injection after cooling by the cooling device = the number of FLACC points at the time of subcutaneous injection without cooling) is rejected at a significance level of 5% on both sides, it is judged that the effect of cooling the subcutaneous injection site with the cooling device on pain relief is confirmed.

Details of analyses not described in this protocol will be described in the "Statistical Analysis Plan" prepared by the person responsible for statistical analysis.

　14.4.1 Aggregation of Patient Background Information

Summary statistics (number of cases, mean, standard deviation, minimum, median and maximum) for quantitative variables and number of cases/percentage (%) for qualitative variables are obtained for demographic and other baseline characteristics for each group.

　14.4.2 Efficacy Analysis

Primary Endpoint

The FLACC scores after subcutaneous injection by a third party will be compared between groups by means of an unpaired t-test. For each group, summary statistics of FLACC scores (number of cases, mean, standard deviation, minimum, median and maximum) and 95% confidence intervals of the difference in means will be calculated.

secondary endpoint

The FLACC scores after subcutaneous injection of the attendant will be analyzed in the same manner as the primary endpoint.

　14.4.3 Safety Analysis

For each adverse event/failure that occurs, the number of cases shall be calculated for each event, and the number and percentage of adverse events shall be calculated. Adverse events that are found to have a causal relationship with the study device shall be considered as adverse events, and shall be tabulated in the same manner as adverse events. In addition, a list will be prepared for the reporting of outbreaks of diseases, etc. as defined by the Clinical Research Act.

14.5 Intermediate analysis

Not implemented.

15. changes in research protocols, procedures, or statistical analysis plans, etc.

The following procedures shall be followed when making changes or additions to the following documents in this study. If it becomes necessary to revise the research protocol after the start of this study, the principal investigator shall prepare the revised research protocol and its revision history, and obtain the CRB's opinion. In cases involving changes in the implementation plan, the implementation plan, the CRB's opinion, and the revised or newly prepared documents shall be submitted to the Minister of Health, Labour and Welfare.

If the original statistical analysis plan is substantially changed, the research protocol and statistical analysis plan shall be revised.

The Principal Investigator shall keep the Principal Investigator and others up-to-date and share necessary information related to this research.

16. Data Management

16.1 Preparing a case report form (CRF)

　The principal investigator and others will prepare a case report form (CRF) using EDC (REDCap introduced in our hospital) for each research subject.

　If the CRF is changed or modified, the reason for the change or modification and the history of the change or modification shall be recorded.

16.2 Identification of Source Materials

　The following documents and other materials will be used as source documents in this study.

(1) Records of TAK-01 use on research subjects

(2) Medical records, nursing records, clinical laboratory data and imaging data, eligibility verification forms, forms prescribed in research protocols and various procedure manuals, and records entered directly into case report forms (electronic data entry system)

(3) Documents or records required under the Clinical Research Act pertaining to this research

16.3 Direct Access to Original Documents

　The Principal Investigator and the site will provide direct access to all study-related records, including source documents, during monitoring and CRB and regulatory investigations related to the study.

17. Quality control and quality assurance

17.1 Monitoring

Monitoring shall be conducted and procedures followed in accordance with the "Procedures for Conducting Monitoring.

17.2 Conducting audits

The principal investigator, the research physician, and the person in charge of statistical analysis will not conduct an audit in this study because they do not have any financial interest in Mie Chemical Industries, Ltd. and TANAC Corporation, and because they believe that there will be no invasion in this study and very little disadvantage to the research subjects.

17.3 Control of Nonconformities

17.3.1 Definition of nonconformity

　　　　Noncompliance with the Clinical Research Act, research protocols, procedures, etc., and falsification or fabrication of research data. Among noncompliance, "serious noncompliance" is defined as those that affect the human rights and safety of clinical research subjects as well as the progress of the research and the reliability of the results. For example, non-compliance with selection/exclusion criteria, discontinuation criteria, concomitant use of prohibited therapies, etc., does not include failure to comply with the research protocol in order to avoid immediate danger to the subjects of the clinical research or for other unavoidable medical reasons.

17.3.2 Reporting of nonconformities

When the principal investigator becomes aware of a nonconformity, he/she shall promptly report it to the administrator of the implementing medical institution and notify the principal investigator of it.

If a "serious nonconformity" is identified among nonconformities, the principal investigator will promptly obtain the opinion of the CRB using the Uniform Form 7 and provide information to the other principal investigators.

18. Compliance with the Clinical Research Act and the Declaration of Helsinki, etc.

This research will be conducted in compliance with the Declaration of Helsinki, the Clinical Research Act, the Conflict of Interest Management Plan for this research, and other relevant laws and regulations.

19. Consideration for the human rights, safety and disadvantages of research subjects

19.1 Consideration for human rights (protection of personal information)

　All persons involved in this research shall comply with applicable laws, ordinances, and regulations regarding the protection of personal information of research subjects. They will also follow the procedures for handling personal information protection at the implementing medical institution. When providing case registration forms and case report forms, etc. outside the implementing medical institution, the principal investigators and subinvestigators shall use the research subject identification code assigned at the time of registration. Information (name, date of birth, address, etc.) that would allow persons outside the implementing medical institution to identify research subjects will not be included. At the start of video recording, the subject is identified by displaying the research subject identification code on a piece of paper, etc., followed by silent imaging during the examination. Although masking cannot be applied to facial images from the perspective of conducting the evaluation, the video data will be provided to the FLACC scale evaluator by the implementing institution, and the FLACC scale evaluator will use the video data for this purpose only, and will comply with the Personal Information Protection Law and other applicable laws, regulations, and rules for handling personal information at Kobe City Medical Center General Hospital. After the retention period, the data will be properly disposed of in accordance with the relevant laws and regulations. Secondary use of video data will not be made.

In any case, a letter of appointment will be issued, the number of persons in charge will be limited, and the research will be conducted appropriately in accordance with the Personal Information Protection Law, the regulations for handling personal information at Kobe City Medical Center General Hospital, and other applicable laws, regulations, and other rules and regulations.

　When publishing the results of this research, sufficient care will be taken to ensure that individual research subjects cannot be identified, but even then, no videos will be published.

19.2 Benefits and Disadvantages to Research Subjects

19.2.1 Projected benefits

　It is expected that participation in this study will alleviate pain caused by immunization.

19.2.2 Anticipated disadvantages (burdens and risks)

　A possible disadvantage of participating in this study is frostbite for the cooling group, but this can be prevented by carefully observing the skin while the physician is applying the skin contact. In addition, since everything from obtaining consent to using TAK-01 will be done on the same day, it is possible that the time spent at the medical institution may be somewhat longer than in the case of non-participation in the study.

19.3 Safety and Adverse Considerations

　Exclusion criteria were used to avoid inclusion of study subjects who were chirping heavily or had perceptual dullness before the injection was administered, to minimize the risk of delay in detecting abnormalities in study subjects. The researcher will carefully observe the study subjects.

20. How to explain to research subjects and obtain their consent

20.1 Subjects for which consent is obtained

　Since the research subjects are preschool children between the ages of 3 and 6 at the time consent is obtained, written consent will be obtained on behalf of the research subjects, such as from their guardian who is their attendant.

20.2 Procedures for obtaining consent from research subjects

　Since the research subjects are preschool children between the ages of 3 and 6 years old at the time of obtaining their consent, we will not obtain written consent from the research subjects themselves, but will explain the research to them using plain language and confirm their willingness to participate as much as possible.

20.3 Procedures for obtaining consent from a surrogate

　Consent must be obtained from a substitute consenter because the research subjects are preschool children between the ages of 3 and 6 years old at the time consent is obtained. The surrogate consentor must be a person within the third degree of kinship of the research subject, a attendant for immunizations, and someone who can monitor the research subject's condition on a daily basis. The principal investigator will fully explain this research to the surrogate using an explanatory document, and obtain written consent from the surrogate to participate in this research. When obtaining consent, the potential surrogate will be given sufficient time and opportunity to ask questions and to answer questions from the surrogate to enable him/her to decide whether or not to participate in the research. The principal investigator who has provided the explanation will confirm that the prospective substitute consents based on a full understanding of the contents of the explanation document. The principal investigator and the intended surrogate will each date and sign the consent form.

　The principal investigator shall deliver a copy of the signed consent form to the surrogate consentor together with the explanation document, and the original consent form shall be kept at the medical institution concerned.

20.4 Explanatory Notes for Substitute

The principal investigator shall prepare a written explanation that includes the following information

(1) Introduction

(2) Clinical research

(3) Reasons for requesting participation in clinical research

(4) Necessity and purpose of clinical research

(5) Methods of clinical research

(6) Contents of survey items

(7) The number of people participating in the clinical research and the duration of the research

(8) Anticipated benefits and disadvantages

(9) In the event of health problems during clinical research

(10) Protection of personal information and access to medical records

(11) Expenses and honoraria related to research

(12) Regarding not being disadvantaged even if one does not consent to clinical research

(13) Discontinuation of treatment after consent

(14) Method of storage and use of data, etc. and storage period

(15) Browsing of documents

(16) Handling of Clinical Research Results

(17) Attribution of Intellectual Property Rights

(18) Clinical research organization and funding sources, and conflicts of interest

(19) What you are expected to observe during the period of participation in clinical research

(20) Contact information for inquiries

21. Compensation for Health Damage

21.1 Indemnification by clinical research insurance, etc.

　The Principal Investigator will obtain research insurance to provide compensation for any health problems that may occur to research subjects as a result of the implementation of this research.

21.2 Non-Clinical Research Insurance Coverage

　No financial compensation other than insurance will be provided.

22. Cost-sharing by research subjects

The TAK-01 to be used in this study will be manufactured jointly by Mie Kogyo Co., Ltd. and Tanak Corporation and will be provided free of charge, so there will be no additional cost to the research subjects (attendants) for participating in the study.

23. Handling and Preservation of Records, etc.

23.1 Preparation of records related to clinical research

In accordance with the Clinical Research Act, the Principal Investigator, etc. will prepare a record of the date and time of use of TAK-01 for each research subject, matters identifying the research subject, matters concerning medical treatment and examination of the research subject, matters concerning participation, and matters necessary for the implementation of the study.

23.2 Record Keeping for Clinical Research

　The principal investigators at each institution shall retain the records related to the implementation of this study and the documents listed below for five years after the completion of this study. After the retention period, they shall be destroyed after making them unidentifiable and unrecoverable.

　Research plan, implementation plan, outline of TAK-01, etc., and source documents

　Explanatory Document and Consent Form for 　Research Subjects and Alternate Consentor

　Summary of the Main Evaluation Item Report, Summary Report, and Summary Report

　Documents received from the CRB pertaining to review and opinion services

　Monitoring Documents

　Contract for the implementation of this research

　Records related to the management of test equipment

　Other documents related to this research that have been prepared by the principal investigator

23.3 Provision of Samples and Information to Other Institutions

Images taken at each institution will be submitted to the FLACC Scale evaluation committee members for judgment. The submission of images and the method of judgment will be described in the "Procedures for the FLACC Scale Evaluation Committee Members", but the image data, which is personal information, will be judged and quantified by the FLACC Scale Evaluation Committee members and used as research data. The principal investigator may publish research data excluding personal information and the results of the safety and efficacy evaluation in a paper, but the images will not be taken outside the evaluation facility. In addition, the results of the research may be provided to Mie Industries, Ltd. and Tanak Corporation, but the images will not be provided.

23.4 Methods of storage and disposal of samples and information

Theresearch data and image data obtained in this study will be processed so that personal information cannot be identified, stored on a hard disk, and stored in a lockable cabinet or similar location. The data will be stored for five years after the completion of this study, and will be disposed of in a non-recoverable manner.

24. Registration of research plan

This study will be registered in the Japan Registry of Clinical Trials (jRCT) based on the recommendation of the International Committee of Medical Journal Editors (ICMJE): (jRCT), based on the recommendation of the International Committee of Medical Journal Editors (ICMJE).

25. Sources of Research Funding and Conflict of Interest Status

25.1 Sources of funding for research

　This research will be conducted with TAK-01 manufactured and provided free of charge by Mie Kogyo and TANAC Corporation. In addition to the in-hospital research grant, this research will be conducted with funding and loan of goods from Tanack Corporation.

25.2 Conflict of Interest Status

　The principal investigator, Ikuo Okafuji, and the principal investigators, subinvestigators, and statistical analysis supervisors at each institution receive funds and goods for their research from Mie Industries, Inc. and TANAC Corporation, and the Conflict of Interest Management Committee at the institution confirms conflicts of interest based on reports from the principal investigators. The conflict of interest management standards and management plan stipulated by the law have been submitted to the CRB for deliberation.

26. attribution of research results and publication of research results

26.1 Attribution of research results

　The results of this research shall belong to the principal investigator.

26.2 Publication of research results

26.2.1 Publication by jRCT

　The Principal Investigator will prepare a "Primary Endpoint Report" within one year after completion of the collection of data pertaining to the primary endpoints for all study subjects. In addition, a "Summary Report" and its summary will be prepared within one year after the completion of data collection for all endpoints.

When the principal investigator prepares the primary endpoint report, the summary report, and its summary, he/she shall obtain the opinion of the CRB and submit it to the administrator of the implementing medical institution without delay. The CRB will also publish them in jRCT within one month from the date of the CRB's opinion.

The principal investigator will provide information to the other principal investigators when the above announcement is made. The other principal investigators who receive the information will promptly report it to the administrator of the site.

26.2.2 Publication in publications and conference activities

　The results of this study will be published through submission to Pediatrics and presentation at the Japan Pediatric Society.

27. Reporting to CRB, etc.

27.1 Periodic Reporting

　The Principal Investigator shall report the following items to the Administrator of the site and the CRB within two months after the expiration of the relevant period every year from the date of submission of the implementation plan to the Minister of Health, Labour and Welfare. When a principal investigator makes a report that falls under this item, he/she shall promptly provide information to the other principal investigators to that effect. In this case, the other principal investigators shall promptly report the contents of such information to the administrator of the implementing medical institution.

(1) Number of research subjects who participated in this study

(2) Outbreak and subsequent progress of diseases, etc. related to this research

(3) Incidence of nonconformity in relation to this research and subsequent actions

(4) Evaluation of the safety and scientific relevance of the study

(5) Status of conflict of interest management

28. Research Organization

See Attachment 1 [Research Implementation Structure].

29. References

1. Eden L, Macintosh J, Luthy K, Beckstrand RL. Minimizing pain during childhood vaccination injections: Improving adherence to vaccination schedules. Pediatr Health Med Ther 2014;5:127-40.

2. Taddio A, Appleton M, Bortolussi R, Chambers C, Dubey V, Halperin S, et al. Reducing the pain of childhood vaccination: an evidence-based clinical practice guideline (summary). CMAJ. 2010;182(18):1989-95.

3. Yamamoto-Hanada K, Futamura M, Kitazawa H, Ohya Y, Kobayashi F, Kusuda T, et al. Relieving pain and distress during venipuncture: Pilot study of the Japan Environment and Children's Study (JECS). Pediatr Int. 2015;57(5):1044-7.

4. Sridharan K, Sivaramakrishnan G. Pharmacological interventions for reducing pain related to immunization or intramuscular injection in children: A mixed treatment comparison network meta-analysis of randomized controlled clinical trials. J Child Health Care. 2018;22(3):393-405.

5. Cassidy KL, Reid GJ, McGrath PJ, Smith DJ, Brown TL, Finley GA. A randomized double-blind, placebo-controlled trial of the EMLA patch for the reduction of pain associated with intramuscular injection in four to six-year-old children. Acta Paediatr. 2001;90(11):1329-36.

6. Hanaoka K, Okubo A. Efficacy and Safety of Lidocaine and Propitocaine Eutectic Mixture Cream (SKA-01) in Patients Scheduled for Venipuncture - A Multicenter, Placebo-Controlled, Randomized, Double-Blind, Parallel Group, Comparative Study. Clinical Medicine. 2015;31(7):683-97 (Japanese).

7. Reducing pain at the time of vaccination: WHO position paper - September 2015. Wkly Epidemiol Rec. 2015;90(39):505-10.

8. Hall LM, Ediriweera Y, Banks J, Nambiar A, Heal C. Cooling to reduce the pain associated with vaccination: A systematic review. Vaccine. 2020;38(51):8082-9.

30. Revision History

| version number | Revision date | Reason for Revision/Contents |
| --- | --- | --- |
| 1.0 | December 11, 2020 | First Edition |
| 1.1 | January 20, 2021 | change corrections |
