# Supplementary material for: Use of a cooling pack to reduce subcutaneous vaccine injection pain in children aged 3–6 years: A single-blind, randomized, parallel-group multicenter study
Source: PLoS One. 2025 Mar 26;20(3):e0318322. doi: 10.1371/journal.pone.0318322 (PMC11940606; doi:10.1371/journal.pone.0318322)
Supplement: S3 File — (DOCX) [file pone.0318322.s003.docx]

**Effectiveness and safety of cooling pack TAK-01**

**for Relieving Pain at subcutaneous injection site in young children**

**Statistical Analysis Plan**

Abbreviation: TAK-01

Principal Investigator:

Department of Pediatrics, Kobe City Medical Center General Hospital

Ikuo Okafuji, PhD.

Statistical Analysis Manager:

Translational Research Center for Medical Innovation

Foundation for Research and Innovation at Kobe

Tatsuo Kagimura PhD.

Creation and modification history

| Version number | Creation-day | Author | Reason for change |
| --- | --- | --- | --- |
| Ver. 1.0 | October 25, 2021 | Ikuo Okafuji | Create new |

**Table of Contents**

1. Objective ............................................................................................................................. 3
2. Research design .................................................................................................................. 3
3. Target number of cases........................................................................................................ 3
4. Assessment item ................................................................................................................... 3
5. Statistical analysis ............................................................................................................... 4
   1. Interim analysis
   2. Basis for setting target number of cases
   3. Significance level used
   4. Handling data
   5. Selection of clinical research subjects for analysis
      1. Definition of the largest analysis population (FAS)
      2. Definition of the target population (PPS) conforming to the research protocol
      3. Definition of safety analysis focal population (SAF)
6. General matters …………………………………………………………………………… 5
   1. Summary of evaluation items
   2. Confidence interval, p-value
   3. Time of statistical analysis
   4. Software used for statistical analysis
7. Statistical analysis items and methods ……………………………………………………. 6
   1. Breakdown of subjects
   2. Breakdown of analysis Population
   3. Discontinued subjects
   4. Dropout subjects
   5. Subject Background
   6. Primary Endpoints
   7. Secondary Endpoints
   8. Safety Assessment
   9. Search Analysis

**1 Objective**

This statistical analysis plan describes the details of the analysis methods of the research protocol "Study of the Effectiveness and safety of cooling Pack TAK-01 in relieving pain at subcutaneous injection site in young infants" Version 1.1 (prepared on February 1, 2021).

**2 Research Design**

Multicenter, two-arm, single-blind, randomized, parallel-group study

**3 Target Number of Cases**

60 cases for efficacy analysis

**4 Assessment Item**

| Primary  Endpoint | FLACC scale evaluation after immunization by FLACC scale evaluation committee member |
| --- | --- |
| Secondary  Endpoint | FLACC scale evaluation after immunization administered by child’s attendant  Occurrence of adverse events |

**5 Statistical Analysis**

**A) Interim Analysis**

No interim analysis will be performed in this study.

**B) Basis for setting target number of cases**

Target number of cases: 60 cases for efficacy analysis

Rationale: For the primary endpoint of FLACC, a two-sample t-test, assuming an effect size of 0.9, a risk rate of 5% (two-sided) and a power of 90%, 27 patients per group would be required. Assuming a dropout rate of about 10% due to inadequate videotaping, 30 cases per group were used.

**C) Significance level used**

When the null hypothesis (FLACC scores at the time of subcutaneous injection after cooling with the cooling pack = FLACC scores at the time of h subcutaneous injection without cooling) is rejected at a significance level of 5% on both sides, it is judged that the effect of cooling the injection site with the cooling device on pain relief is confirmed.

**D) Handling of data**

1. Handling of data

If deviations from the study protocol are found, the impact of the deviations on the efficacy evaluation of the study protocol will be assessed by the principal investigator and the statistical analyst before the database is fixed at the end of the study.

1. Handling of outliers

For outliers, consider how to handle them before conducting statistical analysis.

1. Handling of discontinued research cases

Data collected up to the time of discontinuation of the study will be used as research data. However, it will not be used when there is a withdrawal of consent for the use of data from the research subject.

**E) Selection of clinical research subjects for analysis**

1. Definition of the largest analysis population (FAS)
   The population of study subjects enrolled in the study, for whom subcutaneous injections were administered and at least one efficacy endpoint was measured, will be the largest population for analysis (FAS).
2. Definition of the target population (PPS) conforming to the research protocol

The population of patients included in the FAS who have no significant study protocol violations will be the population of subjects (PPS) who are compliant with the study protocol. Significant study protocol violations will be defined by the principal investigator and the statistical analyst by database fixation after completion of the study.

1. Definition of Safety Analysis Population (SAF)

All patients enrolled in this clinical study and treated with TAK-01 will be included in the analysis.

**6 General Matters**

**A) Summary of evaluation items**

Summary statistics (number of cases, mean, standard deviation, minimum, median and maximum) for quantitative variables and number of cases/percentage (%) for qualitative variables are obtained for demographic and other baseline characteristics for each group.

**B) Confidence interval, p-value**

Two-sided 95% confidence intervals. The p-values are rounded to the third decimal place. However, values rounded to the third decimal place should be displayed to the third decimal place.
If the value is 0.000, display <0.001.

**C) Timing of statistical analysis**

When the period for collecting data pertaining to the primary endpoint has ended, the data pertaining to the primary endpoint shall be fixed and the analysis shall be conducted after the data are fixed. For all endpoints other than the primary endpoint, the analysis will be conducted after the period for collecting data on all endpoints has ended and the data are fixed.

**D) Software used for statistical analysis**

Use JMP14.0.

1. **Statistical analysis items and methods**
2. **Breakdown of subjects**

The breakdown of subjects is shown in the figure.

1. **Breakdown of analysis Population**

[Analysis population: cases in which consent was obtained]

For each analyzed population (FAS, PPS, SAF), the frequency will be tabulated for each group for acceptance or rejection.

1. **Discontinued subjects**

[Analysis population: cases in which consent was obtained]

The frequency of discontinuation and the reason for discontinuation will be tabulated for each group.

1. **Dropout subjects**

[Analysis population: cases in which consent was obtained]
The frequency of dropouts and reasons for dropouts will be tabulated for each group.

1. **Subject Background**

[Analysis population: FAS, PPS]
Frequency and percentage (%) for qualitative variables and summary statistics for quantitative variables are calculated for each group with respect to age, sex, type of immunization, and presence of sensory insensitivity.

1. **Primary Endpoints**

[Analysis population: FAS, PPS]
For the primary endpoint, summary statistics are calculated for each group, and the difference in means between groups and their 95% confidence intervals are calculated and compared between groups by means of an unpaired t-test.
Primary endpoint: FLACC scale evaluation after immunization by FLACC scale evaluators

1. **Secondary Endpoints**

[Analysis population: FAS, PPS]
The secondary endpoints will be analyzed in the same manner as in "F) Primary endpoints," with FAS as the primary analysis and PPS as the secondary analysis. Secondary endpoints: FLACC scale evaluation after immunization by attendant , Occurrence of adverse events

1. **Safety Assessment**

[Analysis population: SAF]
The number of cases of adverse events/failures that occur shall be calculated for each event and each group, and the number and percentage of adverse events shall be calculated. Adverse events that are found to have a causal relationship with the study device shall be considered as adverse events, and shall be tabulated in the same manner as adverse events. In addition, a list will be prepared for the reporting of the occurrence of diseases, etc., as stipulated in the Clinical Research Act.

1. **Search Analysis**

not applicable
