# Supplementary material for: Use of a cooling pack to reduce subcutaneous vaccine injection pain in children aged 3–6 years: A single-blind, randomized, parallel-group multicenter study
Source: PLoS One. 2025 Mar 26;20(3):e0318322. doi: 10.1371/journal.pone.0318322 (PMC11940606; doi:10.1371/journal.pone.0318322)
Supplement: Attachment — Submitted filename: Response_to_Reviewers.docx [file pone.0318322.s004.docx]

**Dear Academic Editor,**

Thank you for the opportunity to revise our manuscript (PONE-D-24-27274). We greatly appreciate the constructive feedback provided by the reviewers, which has helped us to significantly improve the clarity and rigor of our study. Please find our point-by-point responses to each of these comments below:

**Reviewer 1:**

1. **Reviewer Comment**: *“The rationale for not employing the Wilcoxon test for sample size calculation remains unclear.”*

**Response**: As you pointed out, the primary analysis of this study was planned to involve a comparison between groups using the Wilcoxon signed-rank test for the FLACC score. Thus, the sample size calculation for this study would ideally have been performed using the Wilcoxon rank-sum test. However, at the time of planning the study, we were unable to identify a common language effect size based on the U statistic required for the Wilcoxon rank-sum test of the FLACC score in the literature. Consequently, we had no choice but to use an unpaired t-test for the sample size calculation. This information has now been included in the main text (Lines 193-201)

2. **Reviewer Comment**: *“For assessing reliability, it would be more appropriate to utilize either Spearman’s rank correlation coefficient or Cohen’s kappa statistic.”*

**Response**: In accordance with your recommendation, we have now revised Table 4, changing the correlation coefficient to Spearman’s rank correlation coefficient to better assess the reliability. The corresponding results have been updated in the manuscript (Table 4, starting line 257).

3. **Reviewer Comment**: *“Figure 4: The cooling group has fewer than 15 data points.”*

**Response**: Thank you for pointing out the issue with Figure 4. As noted, there was an error in the previous version of the figure; this has now been corrected. Indeed, there were 15 cases in which both the x-axis and y-axis scores were zero. In the previous submission, these data points were not accurately represented. To address this, we have now recreated the figure using a bubble chart, which correctly reflects all cases.

**Reviewer 2:**

1. **Reviewer Comment**: *“The brand names of the vaccines used in the study should be specified.”*

**Response**: We have now added the specific brand names of the vaccines used in this study to ensure clarity (Lines 116-122).

2. **Reviewer Comment**: *“The terminology in the manuscript should be consistent.”*

**Response**: We have carefully reviewed the manuscript to ensure consistency in terminology, particularly in our description of the age range of participants, which focuses on children aged 3-6 years.

3. **Reviewer Comment**: *“The introduction should clearly state the objectives of the study.”*

**Response**: We have now revised the introduction to more clearly define the study objectives, and have moved the FLACC scale table to the Methods section (Table 1, starting line 174).

4. **Reviewer Comment**: *“The limitations of the FLACC scale in younger children should be acknowledged.”*

**Response**: We agree with your point; we have now added a discussion about the potential limitations of the FLACC scale, particularly in children as young as 3 years old (Line 322-326).

**Reviewer 3:**

1. **Reviewer Comment**: *“The advantage of the current cooling method over topical anesthetics should be discussed.”*

**Response**: We have now expanded the Discussion to include a discussion comparing the cooling pack method to topical anesthetics, including both advantages and disadvantages (Lines 297-306).

2. **Reviewer Comment**: *“A comparison of the price between the cooling pack and topical anesthetics would be beneficial.”*

**Response**: We have now included a comparison of the costs between the cooling pack and topical anesthetics in the Discussion section (Lines 303-305).

**Additional Revisions Based on Reviewer and Editor Comments:**

- We have now structured the abstract according to journal guidelines.
- P-values have now been included in the results section of the abstract.
- Additional detail on the inclusion/exclusion criteria has been provided, and the age range has been added to the title.
- We have confirmed the normality of variables and clarified the format for median and interquartile range (IQR) reporting.

**Additional Comments**:

1. **Reviewer Comment**: *“The delay in submitting the study, conducted three years ago, should be explained.”*

**Response**: We appreciate your understanding regarding this delay. Indeed, several factors contributed to the time taken for submission, including our unfamiliarity with the submission process and prolonged response times from previous journals. Furthermore, our clinical workload, which has been exacerbated by staff shortages, limited the time available for writing. Despite these challenges, we remain committed to publishing this study, as we believe it will significantly benefit children by alleviating injection-related pain.

2. **Reviewer Comment**: *“The inclusion and exclusion criteria need more detail, especially regarding skin diseases, which could impact the effect of the cooling pack.”*

**Response**: We considered the potential impact of skin diseases on the cooling pack’s efficacy. However, vaccinations are typically not administered to areas affected by significant skin conditions that may interfere with the cooling pack’s effect. Therefore, we did not find it necessary to explicitly include this in the inclusion/exclusion criteria. If further clarification is required, we will be happy to provide additional details.

We hope that these revisions will address the reviewers' comments satisfactorily. We believe these changes have further improved the quality and clarity of our manuscript, and we look forward to your feedback.

Sincerely,

Ikuo Okafuji

Corresponding Author
Kobe City Medical Center General Hospital
Email: [okafuji@kcho.jp](mailto:okafuji@kcho.jp)
