# Supplementary material for: Use of a cooling pack to reduce subcutaneous vaccine injection pain in children aged 3–6 years: A single-blind, randomized, parallel-group multicenter study
Source: PLoS One. 2025 Mar 26;20(3):e0318322. doi: 10.1371/journal.pone.0318322 (PMC11940606; doi:10.1371/journal.pone.0318322)
Supplement: Attachment — Submitted filename: Response to reviewers_20241229.docx [file pone.0318322.s005.docx]

**Rebuttal Letter**

**Title of Manuscript:** Use of a cooling pack to reduce subcutaneous vaccine injection pain in children aged 3–6 years: A single-blind, randomized, parallel-group multicenter study
**Manuscript ID:** PONE-D-24-27274
**Corresponding Author:** Ikuo Okafuji

Dear Dr. Seyedeh Yasamin Parvar, M.D., M.P.H.,

We sincerely appreciate the opportunity to revise our manuscript, and would like to thank both you and the reviewers for the thoughtful and constructive comments. We have revised the manuscript to address all of these comments, which have greatly enhanced its clarity and quality. Please find below a point-by-point response to these comments, indicating the specific changes made and referencing the corresponding line and page numbers in the revised manuscript.

**Point-by-Point Responses**

**General Editorial Comments**

**Comment:** Provide a point-by-point response to the comments in the same order as they were provided. Indicate the specific parts of the manuscript where changes were made, with detailed references to line and page numbers.
**Response:** We have followed this guideline throughout the rebuttal letter. Each response is organized sequentially, and includes specific references to the revised manuscript.

**Minor Revisions**

1. **Typographical Issues**
   **Comment:** Ensure that all typographical issues mentioned previously have been resolved throughout the manuscript.
   **Response:** We have reviewed the entire manuscript to ensure that all such typographical errors have been resolved.
2. **Use of "Figure"**
   **Comment:** Replace "fig" with "figure" in full and bold this term consistently in the text.
   **Response:** All instances of "fig" have been replaced with "**figure**" throughout the manuscript. Examples can be seen on Page 6, Line 108 and 117

**Specific Revisions Requested by the Editor**

3. **FLACC Scale**
   **Comment:** Using only the FLACC scale may not provide a comprehensive view.
   **Response:** We agree that using only the FLACC scale may not provide a sufficiently comprehensive view. While the FLACC scale was chosen for its suitability for assessing pain in young children, we recognize its limitations in capturing subjective patient experiences such as fear and anxiety. We have added a discussion of this limitation and the potential benefits of incorporating other tools, such as the Children's Fear Scale, in future studies [Page 17, Line 325-329]
4. **Cooling Pack Reference**
   **Comment:** Provide references for this part in the introduction: "A gel-based cooling pack, which maintains its softness even when frozen, may provide a less intimidating alternative for pediatric patients; thus offering a more comfortable and effective pain-relief method during vaccination."
   **Response:** We acknowledge that the original statement lacked supporting references. To address this, we have revised the text to clarify that the described benefits of gel-based cooling packs are based largely on anecdotal observations in clinical practice rather than systematic evidence. Additionally, we have noted the requirements for further studies to substantiate this impression. [Page 5, Line 80-84]
5. **Delay in Submission**
   **Comment:** Explain the delay in submitting the study, conducted three years ago, in the "limitations."
   **Response:** In response to your comment, we have added a brief explanation to the "Limitations" section clarifying that logistical challenges and prioritization of clinical duties contributed to this delay. [Page 17, Lines 329-332]

**Statistical and Methodological Revisions**

6. **Normality of Variables**
   **Comment:** Confirm whether the normality of variables was assessed and specify this in detail in the statistical part of the methods section.
   **Response:** We decided in advance in the protocol that we would conduct a non-parametric test because we thought that the distribution of FLACC scores would not follow a normal distribution, and we did not conduct a non-parametric test after examining the normality of the data obtained, retrospectively.

**Discussion Section Revisions**

7. **First Sentence of the Discussion**
   **Comment:** Rewrite the first sentence of the discussion for clarity.
   **Response:** The first sentence of the Discussion has been rewritten as follows: "This is the first study to reveal that applying local cooling prior to vaccination can alleviate pain associated with vaccination in young children aged 3–6 years." (Page 15, Line 272)
8. **Baseline Anxiety**
   **Comment:** I couldn’t find answer and revisions for this comment. If the focus is on "childhood anxiety and fear of pain," baseline anxiety should be measured and controlled for, as this can influence the results.
   **Response:** We acknowledge that baseline anxiety can significantly impact a child's perception of pain during vaccination. In the present study, we employed randomization to minimize the impact of baseline differences, including anxiety, between the groups. While we did not specifically measure baseline anxiety, randomization ensured that potential differences in baseline anxiety are distributed evenly across the groups, thereby reducing their influence on outcomes. We have added this limitation and suggested that future studies incorporate baseline anxiety measurements. [Page 17, Line 321-325]
9. **Updated Literature**
   **Comment:** Update the manuscript with recently published studies to reflect the current state of research.
   **Response:** We have included references to four recently published studies (References 12, 13, 26, and 27) to better reflect the current state of research.
10. **Cost Reference for Cooling Packs**
    **Comment:** Provide references for this part in the discussion: "While cooling packs are reusable and cost-effective in the long term, they require upfront investment and preparation by hospitals, and the cost may fall on families if purchased individually (3,200 yen per unit)."
    **Response:** We acknowledge that the statement in question lacked supporting references. To address this, we have clarified that the cost-effectiveness of cooling packs has not been rigorously investigated, and that the statement reflects general observations rather than specific research findings. [Page 16, Line 307- Page17, L311]

**Conclusion Section**

11. **Avoiding References**
    **Comment:** Avoid using references in the conclusion.
    **Response:** We have removed the reference from the conclusion section and rephrased the text to summarize the key findings concisely. [Page 18, Lines 338-342]

We hope that these revisions address all concerns raised by the reviewers and editorial team. Please do not hesitate to contact us for further clarification or additional revisions. We look forward to your feedback.

Sincerely,
Ikuo Okafuji
Kobe City Medical Center General Hospital
